# Knowledge, Awareness, and Attitudes about Research Ethics among Dental Faculty in the Middle East: A Pilot Study

**DOI:** 10.1155/2011/694759

**Published:** 2011-07-03

**Authors:** Hadir F. El-Dessouky, Amr M. Abdel-Aziz, Chadi Ibrahim, Malini Moni, Reham Abul Fadl, Henry Silverman

**Affiliations:** ^1^Faculty of Dentistry, King Abdulaziz University, Jeddah 21589, Saudi Arabia; ^2^Faculty of Dentistry, Ain Shams University, Cairo 11566, Egypt; ^3^Departments of Medicine and Epidemiology & Public Health, University of Maryland School of Medicine, Baltimore, MD 21201, USA; ^4^Department of Medicine, Johns Hopkins School of Medicine, Baltimore, MD 21201, USA

## Abstract

*Objective*. To assess the knowledge, awareness, and attitudes of dental faculty regarding research ethics and research ethics committees (RECs). *Design*. Through convenience sampling, we distributed a survey to academics at dental faculties at two universities in the Middle East. We used descriptive, chi-square, and logistic regression statistics to analyze the data. *Results*. Our response rate was 62.5%. A large majority (>90%) held positive attitudes towards RECs; however, almost half (44.0%) thought that RECs would delay research. Less than half (36.8%) had received prior training in research ethics, and the average score they achieved on the questions on research ethics was only 40.2%. Most (>90%), however, were favorable towards research ethics education. Finally, some faculty held attitudes regarding certain research ethics practices that were not optimal. *Conclusions*. We conclude that among the dental faculties participating in our study, there is broad-based acceptance of RECs and training in research ethics, while there are knowledge gaps in research ethics. We recommend further studies to determine the generalizability of our findings to other institutions.

## 1. Introduction

Medical research has increased greatly in many developing countries during the recent decade, motivated by the need to improve health in these countries [1]. Since medical research involves human participants, such research needs to be guided by fundamental ethical principles to ensure the protection of their rights and welfare. Furthermore, international standards mandate the review of research by research ethics committees (RECs) [2, 3]. 

However, research regulations do not exist in many developing countries, and commentators have expressed concerns regarding the extent of individual and institutional research ethics capacity, including the existence of functioning ethics review systems [4, 5]. Accordingly, several studies have demonstrated that research ethics review is not optimal in the developing world, including the Middle East. For example, Abou-Zeid and colleagues found that 28% of researchers in the Middle East Region did not obtain ethical clearance for their research proposals submitted for funding [6, 7]. In many of these proposals, there were no plans to obtain informed consent. These investigators also showed that many basic elements of informed consent were omitted from the submitted informed consent forms [8]. 

Concerns have also been expressed regarding the capacity building efforts for RECs [9] as well as the challenges that prevent the optimal functioning of RECs [10–14]. For example, Sleem and colleagues showed that barriers to the effective functioning of RECs in Egypt include insufficient training of members, lack of diverse membership, and limited resources [10]. Studies from other developing countries have shown similar findings. [11–14]. Less than optimal functioning RECs have been highlighted by recent research-related scandals with occasionally tragic consequences [15, 16]. 

These results demonstrating less than optimal individual and institutional research ethics capacity may be explained by the relative novelty of research ethics regulations and the recent requirement of ethics review of research in the developing world, including the Middle East. As such, little is also known regarding academics' attitudes towards RECs, their practices in research ethics (e.g., informed consent), and training opportunities in research ethics. Recently, Asem and colleagues assessed the knowledge and attitudes of the faculty at Cairo University towards research informed consent [17]. Their results showed that many academics lacked training in research ethics and that their attitudes towards several practices in the research setting were not optimal. However, these investigators also showed acceptance of the faculty towards the establishment of RECs and a desire for educational programs in research ethics. 

The field of dentistry is committed to ongoing research investigating the causes and treatment of dental diseases and adheres to the same ethical standards embraced by the fields of medicine [18]. However, little research has investigated the attitudes of dental faculty towards concepts of research ethics, including the acceptability of RECs and their desire for training in research ethics. Recently, commentators have expressed concerns in dental research related to aspects of scientific misconduct [19, 20]. Accordingly, our objectives were to assess the knowledge, awareness, and attitudes of dental faculty regarding RECs and training in research ethics, as well as potential independent variables associated with our findings. Our results will help institutional officials understand better how well RECs are accepted in their institutions and also help them develop relevant educational programs in research ethics directed towards dental faculty.

## 2. Methods

### 2.1. Study Design

 We conducted a cross-sectional survey study performed during the period between April and June 2007.

### 2.2. Study Participants

 We recruited members of the dental faculty (demonstrators, assistant lecturers, lecturers, assistant professors, associate professors, and professors) at King AbdulAziz University (KAU), Jeddah, Saudi Arabia and at Ain Shams University (ASU), Cairo, Egypt. The roles of these faculty types differed slightly between the two universities. We categorized the different faculty types in the following groups.

#### 2.2.1. Junior Faculty

Defined as faculty with a status of demonstrators or assistant lecturers at ASU and those with a status of demonstrators or lecturers at KAU. Demonstrators at both ASU and KAU cared for patients under supervision, worked on their research projects for their master's theses, and assisted upper level faculty members in students' clinical sessions. Assistant lecturers at ASU and lecturers at KAU held similar academic roles (the rank of assistant lecturers did not exist at KAU): both had obtained their master's degree, worked on their research projects for their PhDs, and attended all students' clinical sessions.

#### 2.2.2. Mid-Level Faculty

Defined as faculty with a status of lecturers and assistant professors at ASU and a status of assistant and associate professors at KAU. Lecturers at ASU and assistant professors at KAU held similar academic roles: both faculty types had obtained their PhDs, were cosupervisors for students' theses, worked on their research projects for their promotions, and gave lectures to undergraduate students. Assistant professors at ASU and Associate Professors at KAU held similar academic roles (the rank of associate professor did not exist at ASU): both worked on their research projects for their promotions, supervised theses, and gave lectures to both under- and postgraduate students.

#### 2.2.3. Professors

Defined as faculty who achieved the status of professors. At both universities, Professors conducted and supervised research projects/theses and gave lectures to both under- and postgraduate students. 

Ain Shams University and King AbdulAziz University are among the most important universities in their respective countries. The Faculty of Dentistry at Ain Shams University was established in 1996 [21]. King AbdulAziz University was founded in 1967 and its Faculty of Dentistry and its four departments (Oral Basic and Clinical Sciences, Oral/Maxillofacial Rehabilitation, Preventive Dental Sciences, and Conservative Dental Sciences) were established in 1985 [22].

### 2.3. Sampling Method

This study used a sample of convenience. We distributed 100 surveys to faculty members at both KAU and ASU. At King AbdulAziz University, one of the coauthors H. F. El-Dessouky, distributed the surveys by placing them into the mailboxes of the faculty in the Dental School. At Ain Shams University, another coauthor R. A. Fadl, distributed the surveys by approaching faculty in their offices. At both universities, faculty was asked to return the surveys back anonymously by placing it in a general mailbox in the Department's Office.

### 2.4. Data Collection Tool

We developed a questionnaire based on our study objectives, taking guidance from the previous literature regarding research ethics in the developing world. The study tool consisted of several parts. The first part collected demographic information of the participants: age, gender, academic position, prior participation in human subjects research (e.g., research involving human subjects and/or human biological samples), number of research projects involved in, and prior training in research ethics (e.g., having attended a course or a workshop in research ethics). We did not ask for any details regarding any courses or workshops the faculty had attended. 

The second part of the survey assessed the participants' self-awareness of research ethics principles and functions of research ethics committees. Specifically, participants were asked the following two questions.

1. Are you familiar with ethical principles that govern conducting research involving human subjects?
2. Are you fully aware of the functions of ethics committees?

The third part of the questionnaire assessed participants' knowledge in research ethics. The first part of the knowledge section consisted of several case scenarios involving the ethics of clinical research in dentistry and asking the respondents to answer questions based on these cases. These cases are as follows.

**Case 1 (Informed consent describing risks and benefits)**

Thirty patients from the outpatient clinic of the Faculty of Dentistry were enrolled in a study that aimed to evaluate the flexible denture base material as compared with conventional denture base. One of the most serious disadvantages of the resilient denture liners is colonization and infection of the material surface by *Candida albicans*. An oral consent has been taken from the patients without full description of the risks and benefits. Which of the following best describes obligations of informed consent?

- The investigators can conduct the research without any ethical responsibility.
- A written consent with a brief description of the procedures must be taken.
- A full description of the risks and benefits should be stated in the informed consent.
- There is no need for informed consent, as the patients were enrolled from the outpatient clinic.

**Case 2 (Research involving children)**

One hundred children of both sexes, age range from 6 to 15 years, were randomly selected from the outpatient clinic of the Faculty of Dentistry. The examined children will be divided into two groups. One group will have their extensively carious teeth extracted, while the other group will go through pulpotomy in an attempt to keep the tooth as long as possible in their mouth.

- A clear description of the procedure should be explained to the child's parent/guardian.
- An assent (oral approval) should be taken from the child.
- An assent should be taken from the child as well as a written informed consent from the child's parent/guardian.
- No need to have an assent or consent as the children were already enrolled in the outpatient clinic and ready to receive any type of treatment.

**Case 3 (Retrospective research on stored samples originally collected for clinical purposes)**

Fifty patients from the outpatient clinic of the Faculty of Dentistry were diagnosed as having Lichen Planus. Biopsies were taken from the patients after their approval to confirm the clinical diagnosis (patients were not charged any money). A month later, a research on Lichen Planus is planned by the Faculty involving all biopsies that were previously obtained from the patients.

- This research cannot be done without the approval of the patients.
- The biopsies belong to the faculty of dentistry, so no patient approval is needed.
- It is for the researchers to decide whether to take the patient's consent or not.
- It is up to the Dean or head of department to decide what to do with the biopsies without patient's interference.

**Case 4 (Confidentiality in medical research)**

Eighty patients from the outpatient clinic were enrolled in a research. The aim of the research was to differentiate between two different treatment modalities in the management of periodontal intraosseous bony defects.

- Patients' research files should be coded to ensure patients' confidentiality.
- No need for confidentiality as the procedures are common in dental practice.
- It is left to the investigator to decide whether to keep the research data confidential or not.
- The dean or head of the department is the one to decide regarding the provisions of confidentiality.

The correct answer for Cases 1 and 2 is “c,” while for Cases 3 and 4 the correct answer is “a.”

These answers were based on concepts of research ethics drawn from research ethics guidelines.

The second part of the knowledge section consisted of the following two questions.

1. Which of the following are considered guidelines in research ethics?
  - Nuremberg Code,
  - Declaration of Helsinki,
  - Belmont Report,
  - Council of the International Organizations of the Medical Sciences (CIOMS), or
  - all of the above.
2. What do you think is the role of a research ethics committee?
  - review the ethical aspects of the research,
  - determine whether informed consent is needed,
  - review the scientific design of the research,
  - protect the welfare and rights of the subjects in the research, or
  - make research more difficult to perform,
  - other.

Correct answers were “e” for question no. 1 and either (a, b, c, d) or (a, b, and d) for question no. 2. We accepted the latter response as a correct answer to question no. 2, because both universities in this study have scientific committees that review the scientific design of the research, and therefore, it is conceivable that faculty might have thought that their REC does not review the scientific design of the research. The CIOMS guidelines recognize this possibility, as they state in their guidelines that a research ethics committee “must either carry out or arrange for a proper scientific review or verify that a competent expert body has determined that the research is scientifically sound.” [3].

The fourth part of the survey assessed respondents' attitudes regarding research ethics committees. Respondents were required to choose from a 5-point Likert scale ranging from 1 to 5 (1-strongly agree, 2-agree, 3-not sure, 4-disagree and 5-strongly disagree). The fifth part of the questionnaire assessed respondents' attitudes towards certain practices in the conduct of research. These practices included those involved with informed consent, enrolment of vulnerable individuals, confidentiality, and responsible conduct of research. Respondents were required to answer “yes”, “no”, or “uncertain”.

### 2.5. Statistics

We entered data from completed questionnaires into Microsoft Excel and then converted it to SPSS version 13.0 (Statistical Package for Social Sciences, 2009). For purposes of analysis, we collapsed the categories of “strongly agree” and “agree”. We report in percentages the positive responses of the available choices (“yes” as opposed to “no” and “do not know” and the “strongly agree” and “agree” responses for those questions employing a Likert scale). We used chi-square tests to determine, in bivariate analyses, the association of each of the independent variables (gender, academic position, prior ethics training, and prior involvement with research) with each of the main outcome of interest (dependent responses involving knowledge, awareness, and attitudes). We used covariates that were significant in a multivariate logistic regression analysis to determine independent predictors (covariates) of the dependent responses. We report in the text the odds ratios and confidence intervals for those associations that were significant. We set the significance level at a *P* value <.05. 

### 2.6. Ethics Statement

#### 2.6.1. Ethics Approval

 This study was approved by the RECs at King AbdulAziz University, Ain Shams University, and the University of Maryland.

#### 2.6.2. Informed Consent

 Potential participants received a cover letter attached to the survey tool that included the following elements of informed consent: the purpose of the research study, potential benefits and risks, and that participation was voluntary and refusal to participate would not be associated with any academic penalty. To ensure anonymity, the RECs waived the requirement of signed written consent; completion of the survey implied participants' provision of informed consent.

## 3. Results

Of the 100 questionnaires distributed at each university (total of 200), we received responses from 125 individuals. There were 75 surveys from King Abdul Aziz University (KAU) and 50 from Ain Shams University (ASU), representing response rates of 50% and 75%, respectively, and a total response rate of 62.5%. Table 1(a) shows the demographic data of the faculty from the universities, both separately and combined. There were no significant differences between the two universities regarding the demographic data. The combined results showed that there were slightly more women faculty compared to men (55.2% versus 43.2%), and there was an almost even distribution among the different faculty types. Regarding prior research experience, almost three quarters of the respondents from both universities (71.2%) had performed research involving human subjects or human tissue samples or both. Regarding prior ethics training, a minority (36.8%) of the respondents had prior training in ethics, whereas a majority (63.2%) had no such prior training. Of those faculty who received ethics training, 16 (35%) had attended both a course and a workshop, whereas 30 (65%) had attended either a course or a workshop, but not both (data not shown in table). 

Table 1(b) shows the association between gender and academic position with prior research experience (percentage of faculty performing research and number of projects/faculty) and prior ethics training. Research experience was higher with men compared with women, both in terms of the percentage of faculty (85.2% versus 61.2%, *P* < .01) and in the mean number of research projects per faculty (7.56 versus 3.31, *P* < .01). Regarding research experience, while the percentage of faculty performing human subjects research was similar among the different faculty types, the mean number of research projects per faculty was higher for the more senior faculty. Faculty at ASU and KAU performed similar mean number of projects per faculty (5.34 versus 5.20 projects/faculty, *P* = NS; data not shown in table). Regarding prior ethics training, men and women showed similar percentages, while there was a significantly higher percentage of Mid-Level faculty who had prior ethics training (57.5% versus 28.2% and 26.2% for Professors and Juniors, resp., *P* < .01). The percentage of faculty with prior ethics training was similar between those with and without prior research experience involving human subjects (37.1% versus 32.4%, resp., *P* = NS; data not shown in table).

Table 2 shows the respondents' responses regarding their awareness of research ethics principles and the functions of RECs. Less than half of the respondents stated that they were familiar with research ethical principles, and less than a third stated that they were familiar with the functions of RECs. A higher percentage of faculty at KAU compared with those at ASU stated their familiarity with research ethics principles (*P* < .05). Table 2 also shows the association between the responses and demographic (independent) variables. Professors were significantly more likely to state that they were familiar with research ethics principles (*P* < .01); Mid-Level faculty was significantly more likely to state they were familiar with the functions of an REC (*P* < .01). Faculty with “prior ethics training” were significantly more likely to state they were familiar with research ethics principles and with the functions of RECs compared with those who had no such prior training (both *P* < .01). There was a tendency for faculty with prior research experience involving human subjects to more likely state they were familiar with research ethics principles and functions of RECs compared with faculty without such research experience, but these differences did not reach statistical significance.

We performed a multiple logistic regression analysis to determine which independent variables were the strongest predictors of the responses in Table 2. Our analysis showed that “prior ethics training” was a strong predictor for stating a familiarity with research ethics principles (*P* < .001, OR 10.10; 95% CI, 2.45–41.67) and for stating a familiarity with the functions of RECs (*P* < .001; OR 5.95; 95% CI 2.41–14.68). 

Table 3 shows the responses to the case scenarios (items no. 1–4) and questions (items no. 5 and 6) that assess respondents' knowledge in research ethics. More than half of the respondents gave the correct answer for the first case involving an informed consent issue, while less than half of the respondents gave correct answers for the other three case scenarios. A small number of respondents (12.0%) knew the guidelines in research ethics (Item no. 5) and less than a third knew the roles of RECs (item no. 6). Regarding an overall score, the average score was 40.2%; 15.2% of the respondents gave correct answers to at least five of the questions, while approximately half (56.0%) knew the correct answers to at most two of the questions. 

Table 3 also shows the association between the responses and the demographic (independent) variables. Faculty at KAU were significantly more likely to give the correct response to the 4th and 6th items (both *P* < .05). Mid-level faculty were significantly more likely to give the correct answers to several of the knowledge questions (2nd, 3rd, and 6th items; *P* < .01, *P* < .01, and *P* < .05, resp.) compared with the other faculty types. There was a tendency for faculty with “any prior ethics training” or “prior research experience” to more likely give correct answers to all of the questions compared with those without prior ethics training or prior research experience; but these differences only reached statistical significance for item no. 5 for faculty with “prior ethics training” (*P* < .01). We performed a multiple logistic regression analysis to determine which independent variables were the strongest predictors of the responses in Table 3. Our analysis showed that Mid-Level faculty was a strong predictor of knowing the correct responses to the 2nd and 3rd cases; (both *P* < .001; OR 8.62; 95% CI 2.87–25.64; OR 10.75; 95% CI 3.16–37.03; resp.); and that “prior ethics training” was a strong predictor for knowing the guidelines in research ethics (item no. 5; *P* < .001; OR 10.55; 95% CI, 2.60–42.86). 

Regarding the “overall score”, faculty at KAU compared with those at ASU achieved a higher average score (47.0% versus 37.0%, *P* < .01), whereas Mid-Level faculty had a higher average score compared with that obtained by the Professor and Junior faculty (55.8% versus 33.8% and 32.2%, resp., *P* < .01). KAU faculty, Mid-Level faculty, and those with “prior ethics training” were significantly more likely to give correct answers to at least five of the questions (*P* < .01, *P* < .01, and *P* < .05, resp.). Logistic regression revealed that KAU faculty and Mid-Level faculty were strong predictors for knowing the correct answers to at least five of the questions (*P* < .05; OR 4.69; 95% CI, 1.13–19.53; *P* < .01; OR 9.52; 95% CI, 1.66–55.56, resp.). 

Table 4 shows the respondents' attitudes to RECs and research ethics education. Greater than 90% of the respondents agreed that an REC would be helpful, there is a need for an REC in each institution, and that human subject research must be reviewed by an REC. Furthermore, less than 20% believed that ethical review is only necessary for international collaborative research and less than 10% thought that the presence of scientific committees made the existence of an REC unnecessary. However, almost half (44%) thought that RECs would delay research and would make research harder to perform. A large majority of the respondents (greater than 90%) were in favor of research ethics education for postgraduates, investigators, and members of RECs. 

Table 4 also shows the association between these attitudes and the demographic variables. Of note, those “without any prior ethics training” were significantly more likely to think that an REC would be helpful (*P* < .01). Furthermore, Mid-Level faculty and those with “prior ethics training” were significantly more likely to believe that an REC would delay research (*P* < .05). Professors compared with the other faculty were significantly more likely to agree that ethical review of research is only necessary for international research (*P* < .01). Multiple logistic regression analysis showed that none of the independent variables were strong predictors for any of these attitudes.

Table 5 shows the respondents' attitudes toward several practices in research ethics. A significant majority of the respondents (>90%) believed in the need for confidentiality protections of research participants' data (question no.1). A large majority (>85%) also held strong opinions regarding the importance of informed consent, as indicated by their responses to questions no. 2–5. Less than 10% believed that patients should not be told about the risks of research because they may not enroll in the study. However, almost a third of the respondents thought it was not necessary to obtain research informed consent for blood samples that were obtained for clinical tests (question no. 7). Almost 40% of the respondents thought that vulnerable groups such as children and the mentally ill could provide informed consent (question no. 8). A small minority (<10%) of the respondents thought that if surrogates are not available to give informed consent for vulnerable individuals, it would still be proper to enroll such individuals in research (item no. 9). Slightly more than 10% of the respondents thought it is proper to fabricate data to improve the outcome of the research if such an act did not cause harms to patients (question no. 10). 

Table 5 also shows the association between these attitudes and the demographic variables. Of note, men and those with prior research experience were significantly more likely to agree that vulnerable groups could provide informed consent (both *P* < .01). Professors were significantly more likely to believe that “research informed consent is not necessary for blood samples obtained for clinical test” and that it is “okay to fabricate data” (*P* < .01). Multiple logistic regression analysis revealed that “prior research experience” was a strong predictor for agreeing that vulnerable groups could provide informed consent (*P* < .01; OR 4.55; 95% CI 1.54–13.41).

## 4. Discussion

This survey study showed several key findings that should be of interest to educators and policy makers. First, the surveyed dental faculty indicated a high endorsement for the existence of RECs, as most thought that such committees should review research, that they would be helpful, and that they should exist in universities. Previous studies observed similar results regarding the acceptance of RECs among academics in Sudan and Egypt [17, 23]. 

However, while the faculty in this study endorsed the existence of RECs, almost half of them held the opinion that such committees would delay research and make it more difficult to perform research. Commentators from Western countries, where RECs have been in existence for more than twenty years, have recently written about concerns with excessive bureaucratic details that cause costly delays in research approval [24–26]. In our study, respondents who are more likely to harbor the belief regarding delays in research by RECs were Mid-Level faculty and those with prior ethics training. Several reasons might explain these results. First, faculty, in general, might not understand the extent of the processes needed for an adequate review of research. Indeed, less than a third of all of the respondents stated their familiarity with the functions of RECs, underscoring that efforts are needed to enhance faculty awareness of the operations of the RECs. 

Second, the finding that faculty with prior ethics training were more likely to believe that RECs would delay research raises the question as to whether the prior training gave a mistaken impression about RECs by emphasizing the many processes of RECs without stressing the benefits of REC review. Third, that Mid-Level faculty was more likely to believe that RECs would delay research can be explained by them having had more unfavorable experiences with their RECs. Indeed, Mid-level faculty was shown to have conducted more human subjects research projects than the Junior faculty and might have had more interactions with RECs compared with Professors, who probably did the majority of their research when RECs were not in existence. Finally, it is possible that these results regarding the associations between Mid-level faculty and “prior ethics training” with the belief that RECs delay research might represent “false positives”, since these independent variables were not significant on multiple logistic testing. Future qualitative research involving in-depth interviews is warranted to further explore the basis of faculty attitudes regarding the process of research review by RECs. 

Our survey also yielded interesting results regarding the attitudes of the dental faculty towards certain research ethics practices. A large majority of the faculty appears to be aware of the accepted practices regarding confidentiality protections and several aspects regarding the informed consent process. These results contrast with the concerns mentioned regarding informed consent practices by commentators working in Egypt [27]. However, approximately a third of the respondents held the attitude that research performed on blood samples obtained for clinical purposes do not require informed consent. Professors were more likely to agree with this practice compared with the other faculty. Asem and colleagues observed similar results regarding this issue among the faculty at Cairo University [17]. Another concern we discovered regarding informed consent is that almost 40% of the faculty believed that certain vulnerable subjects (e.g., children and the mentally ill) could provide informed consent to participate in research. This result raises the underlying issue of how “informed” is informed consent for subjects who might lack decision making capacity. This result is made more significant by the findings of another study showing that research participants who participated in studies on oral health in Nigeria had poor understanding of several key elements of the informed consent process [28]. Accordingly, we suggest future training efforts for dental faculty to be focused on informed consent issues, including those issues involved with concepts of understanding and vulnerability. 

A small but significant minority (approximately 10%) thought it is permissible to fabricate data and Professors were more likely to hold this opinion compared with the other faculty types. This percentage is similar to various previous studies. For example, Eastwood and colleagues found that approximately 2% of respondents were willing to fabricate data for a grant application or a paper, whereas about 27% would be willing to select or omit data to improve the results [29]. Similarly, a study of biomedical trainees showed that 15% admitted of personal misconduct, and they were willing to fabricate, select, or omit data for publishing a paper or obtaining a research grant [30]. Finally, a recent meta-analysis of surveys involving scientists' self-report of research misconduct yielded a pooled weighted estimate of almost 2% of the scientists replying that they had fabricated, falsified, or modified data at least once, with a range from 0.3% to 4.9% [31]. These numbers reflecting “self-report” are lower than those in the previously mentioned studies reporting what individuals “would be willing to do”, thus highlighting the difference between “perception” and actual “practice”. Finally, Martinson and colleagues also showed that scientists late in their careers admitted to fabrication at rate higher than those early in their careers [32]. Reasons that may account for differences between the different faculty levels regarding fabrication include (a) senior faculty have had more opportunities to engage in misconduct, (b) perceptions of being caught might change during one's career, and (c) senior faculty received their education and work habits at a time when there were different behavior standards. 

Our survey also yielded important results regarding training capacity in research ethics. Specifically, less than half (36.8%) of the 125 respondents from both universities received prior training in research ethics. Furthermore, the overall average score achieved on the knowledge questions was only 40.2%, and only about 15% of the faculty was able to give correct answers to at least five of the six questions. Thus, lack of training and knowledge gaps on research ethics exist among the academic dental faculty. Interestingly, while the variable “prior ethics training” was shown to be an independent predictor for awareness of ethical principles and the functions of RECs, it was not an independent predictor for achieving a high score on the knowledge-type questions. These results raise the issue that current training programs in research ethics might be insufficient. 

Interestingly, Mid-Level faculty compared with the other faculty types were more likely to score well on the knowledge-type questions. Several reasons can be offered to explain these results regarding the Mid-level faculty. First, as shown in Table 1(b), Mid-Level faculty was more likely to have received prior ethics training compared with the other faculty types. However, as explained previously, “prior ethics training” was not an independent predictor for knowledge. But, ethics training coupled with ample research experience might have led these faculty to obtain the amount of theoretical and practical knowledge necessary to answer the questions on our survey. Indeed, Mid-level faculty had a combination of ethics training and research experience (defined by number of projects/faculty) that together was greater than observed for the other faculty types. Alternatively, Mid-level faculty might have had more recent opportunities to travel abroad for their academic education and thus had more exposures with research ethics issues from their experiences at these other universities. Further research should probe for the factors that account for any differences in knowledge gaps between the different faculty types. 

Although knowledge gaps exist in research ethics, our findings showed that all faculty levels were favorable towards research ethics training for postgraduates, investigators, and REC members. Previous studies have had mixed results regarding the effects of ethics education on knowledge. A 3-day workshop in research ethics involving clinicians and scientists in a Nigerian university improved participants' knowledge and application of the principles of research ethics, international guidelines and regulations, and operations of RECs [33]. Other investigators have shown similar findings regarding the effects of ethics training [34–36]. However, other studies have demonstrated the lack of an effect of prior ethics instruction on the extent of research ethics knowledge [37, 38]. Also, previous studies have yielded inconclusive results regarding positive relationships between ethics education and moral conduct [29, 30, 39–41]. These findings do not necessarily call into question the value of ethics education, but rather the quality of the educational experiences, as well as the long-range effects of short training in research ethics. Further research is needed to determine the teaching methods (e.g., face-to-face or distance learning) that are most effective in addressing the existing knowledge gaps in research ethics.

We recognize several limitations to our study. First, our study was based on convenience sampling, thus the professionals who completed the survey may not reflect the awareness, knowledge, and attitudes of the entire membership of the dental faculty of the two universities. Second, this study involved one university in each of two countries in the Middle East, thus further limiting the generalizability of our results. Future studies are warranted to investigate the knowledge, awareness, and attitudes of academics from other faculties in other universities. Third, the knowledge type questions we used in our study do not reflect the broad range of topics in research ethics. However, the questions on the survey represent basic information that academics should be expected to know in the area of research ethics. Finally, we did not obtain detailed information regarding the types of courses and workshops that the faculty had attended, as well as the methods of instructions used in their training.

Despite these limitations, our study reveals important information. First, there appears to be an acceptance of RECs among the faculty. Second, there seems to be an acceptance of the need for education in research ethics among all faculty levels. Since, as revealed in our study, prior research ethics training was not an independent predictor for the extent of knowledge in research ethics, attention should be directed towards the type and extent of training needed to further enhance faculty knowledge on research ethics issues. We therefore recommend further development of educational instruction in research ethics for all university faculty, with special emphasis on vulnerable participants, responsible conduct of research, and the roles and functions of RECs. Recently, educational initiatives have been organized in many regions in the developing world [42, 43]. Such efforts can lead to enhanced knowledge and acceptance of research ethics principles among investigators. Finally, we recommend qualitative studies to further explore the attitudes of faculty towards RECs and certain practices in research ethics.

## Figures and Tables

**Table:** (a)

Characteristic	King Abdul-Aziz (*n* = 75) *n* (%)	Ain Shams (*n* = 50) *n* (%)	Total (*n* = 125) *n* (%)
Gender			
Men	32 (43.8)	22 (44.0)	54 (43.2)
Women	41 (56.2)	28 (56.0)	69 (55.2)
Academic position			
Professor	24 (32.0)	15 (30.0)	39 (31.2)
Mid-level	28 (37.3)	12 (24.0)	40 (32.0)
Junior	23 (30.7)	23 (46.0)	46 (36.8)
Prior involvement with research			
Prior research experience	51 (68.0)	38 (76.0)	89 (71.2)
Research involving human subjects	48 (65.8)	34 (68.0)	82 (66.7)
Research involving biological samples	33 (45.2)	21 (42.0)	54 (43.9)
No prior research involving human subjects/samples	22 (32.0)	12 (24.0)	34 (27.2)
Prior training in research ethics			
Prior training (either workshop/course or both)	29 (38.7)	17 (34.0)	46 (36.8)
No prior training	46 (61.3)	33 (66.0)	79 (63.2)

^+^numbers may not add to 100% due to some individuals declining to answer the question.

**Table:** (b)

Item	Aggregate	Gender		Academic position		
		Men	Women	Prof	Mid-level	Junior
Prior research experience (% of faculty)	71.2	85.2**	61.2	70.3	75.0	71.7
Number of projects/faculty (mean)	5.26	7.56**	3.31	8.34**	5.97	2.61
Prior ethics training (% of faculty)	36.8	38.9	36.2	28.2	57.5**	26.1

*P < .05; **P < .01.

**Table 2:** Awareness of research ethics and research ethics committees: aggregate responses and association between responses and independent variables. Numbers represent percentage of “yes” or “correct” responses.

Item	Aggregate*n* = 125 (%)	KAU*n* = 75 (%)	ASU*n* = 50 (%)	Gender (%)		Academic position (%)			Prior ethics training (%)		Prior research (%)	
				Men	Women	Prof	Mid-level	Junior	Any Ethics Training	None	Any	None
Are you familiar with ethical principles in human subject research?	46.4	54.7*	34.0	51.9	40.6	59.0**	45.0	37.0	69.6**	32.9	50.6	32.4
Are you familiar with the functions of a research ethics committee?	31.2	36.0	24.0	31.5	29.0	33.3	40.0**	21.7	52.2**	19.0	36.0	20.6

*P < .05; **P < .01.

**Table 3:** Knowledge of research ethics: aggregate correct responses to questions and association between correct responses and independent variables. Numbers represent percentage of correct responses.

Question	Aggregate*n* = 125 (%)	KAU*n* = 75 (%)	ASU*n* = 50 (%)	Gender (%)		Academic position (%)			Prior ethics training (%)		Prior research (%)	
				Men	Women	Prof	Mid-level	Junior	Any ethics training	None	Any	None
(1) Informed consent	67.2	73.3	58.0	57.4	73.9	64.1	80.0	58.7	76.1	62.0	70.8	55.9
(2) Research involving children	48.0	54.7	38.0	38.9	53.6**	30.8	72.5**	41.3	56.5	43.0	48.3	44.1
(3) Retrospective research involving tissue samples	35.2	41.3	26.0	48.1**	23.2	17.9	60.0**	28.3	45.7	29.1	39.3	26.5
(4) Confidentiality	49.6	58.7*	36.0	55.6	43.5	38.5	70.0	41.3	58.7	44.3	55.1	32.4
(5) Knowledge of different guidelines in research ethics	12.0	14.7	8.0	14.8	10.1	10.3	15.0	10.9	26.1**	3.8	14.6	5.9
(6) Knowledge of the roles of a research ethics committee	29.3	37.0*	20.0	29.6	29.0	28.2	45.0*	19.5	34.8	27.8	33.7	23.5

Overall Score												
Average Score (%)	40.2	47.0**	30.0	42.0	37.7	33.8	55.8**	32.2	50.3	34.3	42.5	33.8
>80% (5-6 correct)	15.2	21.3**	6.0	14.8	13.0	10.3	30.0**	6.5	23.9*	10.1	14.6	17.6
>50% (3-4 correct)	28.8	36.0	18.0	35.2	24.6	25.6	37.5	23.9	32.6	26.6	31.5	17.6
<50% (0–2 correct)	56.0	42.7	76.0	50.0	62.3	64.1	32.5	69.6	43.5	63.3	53.9	64.7

*P < .05; **P < .01.

**Table 4:** Attitudes regarding research ethics committees (REC) and research ethics education: aggregate responses and association between responses and independent variables. Numbers represent percentages of respondents who “strongly agree” and “agree”.

Item	Aggregate *n* = 125 (%)	KAU *n* = 75 (%)	ASU *n* = 50 (%)	Gender (%)		Academic position (%)			Prior ethics training (%)		Prior research (%)	
				Men	Women	Prof	Mid-level	Junior	Any Ethics Training	None	Any	None
(1) A research ethics committee would be helpful	91.2	92.0	90.0	94.4	88.4	100.0*	80.0	93.5	80.4	97.5**	91.0	91.2
(2) There is a need for a research ethics committee in each institution for ethical review of research	92.0	88.0	98.0	98.1	87.0	94.9	92.5	89.1	97.8	88.6	94.4	85.3
(3) Research with human subjects must be reviewed by a research ethics committee	94.4	90.7	100.0	96.3	92.8	94.9*	100.0	89.1	97.8	92.4	94.4	94.1
(4) Ethical review of research is only necessary for international collaborative research	19.2	14.7	26.0	14.8	23.2	30.8**	10.0	17.4	17.4	20.3*	20.2	17.6
(5) Ethical review of research by an REC is not necessary since there are scientific committees	9.6	10.7	8.0	7.4	11.6	10.3	5.0	13.0	6.5	11.4	5.6	20.6
(6) Ethical review of research by an REC would delay research and make it harder for the researcher	44.0	44.0	44.0	57.4*	31.9	41.0	57.5*	34.8	50.0*	40.5	44.9	44.1
(7) The members of a research ethics committee should receive training in research bioethics	92.8	90.7	96.0	87.0	97.1	94.9	90.0	93.5	100.0	88.6	89.9	100.0
(8) Research ethics should be taught as a mandatory postgraduate module	96.0	94.7	98.0	96.3	95.7	100.0	100.0	89.1	100.0	93.7	95.5	97.1
(9) All investigators should have some training in research ethics	97.6	98.7	96.0	96.3	98.6	97.4	100.0	95.7	100.0	96.2	97.8	97.1

*P < .05; **P < .01.

**Table 5:** Attitudes toward practices in research ethics: aggregate responses and association between responses and independent variables. Numbers represent percentages of respondents who answered “yes”.

Research ethics practice	Aggregate *n* = 125 (%)	KAU *n* = 75 (%)	ASU *n* = 50 (%)	Gender (%)		Academic position (%)			Prior ethics training (%)		Prior research (%)	
				Men	Women	Prof	Mid-level	Junior	Any Ethics Training	None	Any	None
(1) There should be measures to protect patient data from being accidentally exposed	93.6	94.7	92.0	96.3	91.3	100.0	97.5	84.8	95.7	92.4	92.1	97.1
(2) Patients should be informed about the full details of the research including all risks and benefits	90.4	85.3	98.0	92.6	88.4	89.7	92.5	89.1	95.7	87.3	92.1	91.2
(3) Informed consent should always be obtained by having patients sign a written form	91.2	88.0	96.0	88.9	92.8	100.0	87.5	87.0	95.1	88.6	87.6	100.0
(4) Informed consent from patients is necessary for use of their biological samples in research	87.2	92.0	80.0	87.0	87.0	89.7	97.5	76.1	87.0	87.3	88.8	88.2
(5) When involving patients in research that presents greater than minimal risk, informed consent must be sought from each patient	91.2	94.7	86.0	92.6	89.9	97.4	97.5	76.1	93.5*	89.9	91.0	91.2
(6) Patients should not be told about potential risks of a study because they may not enroll in the study.	7.2	9.3	4.0	3.7	7.2	10.3	0.0	10.9	2.2	10.1	6.7	8.8
(7) No need to obtain research informed consent for blood samples obtained for clinical tests	32.8	36.0	28.0	30.4	37.0	38.5**	14.3	37.9	23.9	39.2	34.8	32.4
(8) Vulnerable groups such as children and the mentally ill could provide informed consent	39.2	40.0	38.0	53.7**	26.1	41.0	50.0	28.3	41.3	38.0	49.4**	14.7
(9) If no surrogate is available to give informed consent for vulnerable groups, they could still be included	7.2	5.3	10.0	3.7	10.1	12.8	5.0	4.3	6.5	7.6	7.9	5.9
(10) It is okay to fabricate data to improve outcome of research as long as there is no harms to the patients	11.2	10.7	12.0	5.6	15.9*	23.1**	0.0	10.9	13.0	10.1	10.1	14.7

*P < .05; **P < .01.
